# Resting energy expenditure and optimal nutrition in critical care: how to guide our calorie prescriptions

**DOI:** 10.1186/s13054-017-1717-y

**Published:** 2017-06-05

**Authors:** Oren Zusman, Pierre Singer

**Affiliations:** 1 Department of Internal Medicine E, Rabin Medical Center, Beilinson Hospital, Petah Tikva, Israel; 2 Department of General Intensive Care and Institute for Nutrition Research, Rabin Medical Center, Beilinson Hospital, Petah Tikva, Israel; 3 Sackler School of Medicine, Tel Aviv University, Tel Aviv, Israel

We thank Berger et al. [1] for their interesting comment regarding our study [2]. Briefly, they suggest that “feeding progression days” might induce bias so that the administered calories/resting energy expenditure (REE) percentage (% Adcal/REE) we show associated with better outcome is lower than the “true” mean. We agree that for the short-stayers included in the cited studies this remark is pertinent, but we would like to exclude studies based on predictive equations since they lack accuracy and may mislead our understanding. According to clinical practice [3], calories are usually increased progressively to the plateau target, but in addition, calories administered may vary during ICU stay due to interruptions in nutrition administration, making evaluations even more complicated.

Our study, using thousands of measured REEs acquired by indirect calorimetry, was performed from admission, with a local strategy to quickly increase calorie intake to target. Following Berger et al.’s suggestion, we present here a sensitivity analysis, including only feeding days from day 3 and onwards, based on the fact that, from this day, calorie intake didn’t change significantly per day (Fig. 1 in our original study). We still found a significant association with mortality (*p* = 0.003). In addition, we have analyzed patients with more than ten evaluable ICU nutrition days. Figure 1 here shows the original curve along with the respective ones from the sensitivity analysis. The U-shaped curve is preserved, and the “optimal” point of % Adcal/REE after excluding two days is similar (71% after excluding first two days, and 80% including only patients with more than ten evaluable nutrition days). So the first days’ effect is minimal and does not change the study’s message, surely not moving the target to 95–105% as proposed by Berger et al. This does not fit the results reported in a supplemental parenteral nutrition study [4], possibly because it was powered to demonstrate a reduction in morbidity and not in mortality.

**Fig. 1:**
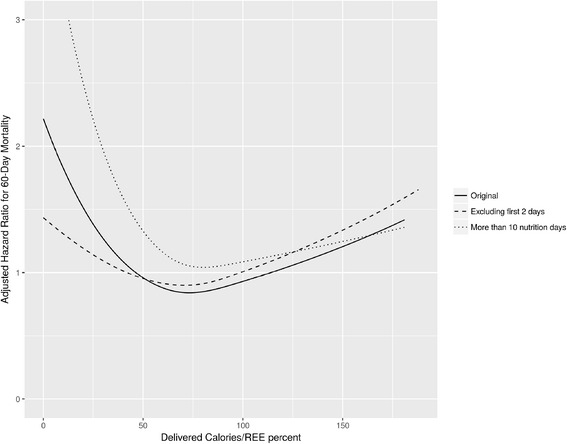
Association of administered calories/REE percentage with mortality in different models

Practically, daily calorie needs and administration cannot be expected to be constant. After reanalysis, our observation still stresses the importance of using % Adcal/REE as measured by indirect calorimetry, as it demonstrated association with reduced mortality. Our take home message remains that our aim should be to target 100% of REE and, due to practicalities of daily care, ultimately achieve 70–80% over the course of the ICU stay. This may serve as a strong basis for further studies.

## Abbreviations

% AdCal/REE: percentage administered calories/resting energy expeniture
ICU: intensive care unit
